# Clinical significance and prognosis of serum tenascin-C in patients with sepsis

**DOI:** 10.1186/s12871-018-0634-1

**Published:** 2018-11-15

**Authors:** Weifang Yuan, Wei Zhang, Xiaofang Yang, Liyuan Zhou, Ziwei Hanghua, Kailiang Xu

**Affiliations:** 1 Department of Intensive Care Medicine, Seventh People’s Hospital of Shanghai University of TCM, No.358 Datong Road, Gaoqiao Town, Pudong New District, Shanghai, 200137 China; 2 Department of Emergency Surgery, Seventh People’s Hospital of Shanghai University of TCM, Shanghai, 200137 China

**Keywords:** Sepsis, Tenascin-C, Mortality, Biomarkers

## Abstract

**Background:**

Tenascin-C is a pro-inflammatory glycoprotein with various biological functions. High expression of tenascin-C is found in inflammation, tissue remodeling, and autoimmune diseases. However, its expression and clinical significance in sepsis remain unclear. This study was designed to investigate the relationship between serum tenascin-C levels and disease severity and prognosis in patients with sepsis.

**Methods:**

A total of 167 patients with sepsis admitted to the ICU were enrolled. Lood samples were collected within 24 h of admission. Serum tenascin-C levels were measured by enzyme-linked immunosorbent assay (ELISA). Follow-up was performed to observe 30-day mortality.

**Results:**

Serum tenascin-C levels were significantly elevated in patients with sepsis compared with non-sepsis controls (*P* < 0.001). Serum tenascin-C levels were higher in nonsurvivors (58 cases) who died within 30 days (34.5%) compared to survivors (109 cases) (*P* < 0.001). In patients with sepsis, serum tenascin-C levels were significantly positively correlated with SOFA scores (*P* = 0.011), serum creatinine (*P* = 0.006), C-reactive protein (CRP) (*P* = 0.001), interleukin-6 (IL-6) (P < 0.001), and tumor necrosis factor α (TNF-α) (*P* = 0.026). Logistic multivariate regression models showed that serum tenascin-C levels were independent contributor of 30-day mortality. Kaplan-Meier curves showed that septic patients with high levels of serum tenascin-C (≥56.9 pg/mL) had significantly higher 30-day mortality than those with lower serum tenascin-C (< 56.9 pg/mL) (*P* < 0.001).

**Conclusion:**

Elevated serum tenascin-C was found in septic patients and associated with severity and poor prognosis.

## Background

Sepsis is a systemic inflammatory response caused by infection and has a high risk of multiple organ dysfunction syndrome (MODS) and death [1]. It is currently believed that severe sepsis causes systemic inflammatory response syndrome (SIRS) and has a compensatory anti-inflammatory response [2]. At the same time, the complex interaction between pro-inflammatory and anti-inflammatory responses determines the outcome of sepsis [3]. Therefore, new biomarkers are required for early diagnosis and prognosis prediction of severe sepsis patients [4].

Tenascins are a group of extracellular matrix glycoproteins that are expressed during the development of multicellular organisms, and are involved in a variety of pathological processes such as inflammation, tissue damage, tumor angiogenesis and metastasis [5]. Tenascin-C is a multi-domain protein linked by disulfide bonds. Obvious expression of tenascin-C can be measured during embryonic development, especially in sites with higher cell turnover such as stem cell niches [6]. The expression of tenascin-C in adults is limited to the site of tissue injury, usually temporary, and the expression level of tenascin-C returns to normal after tissue repair is completed. In contrast, the sustained high expression of tenascin-C is common in inflammation, tissue remodeling, and autoimmune diseases [7].

Infection and injury can induce the expression of tenascin-C, which promotes the body to produce an effective immune response to bacterial lipopolysaccharide (LPS). Tenascin-C enhances the synthesis of pro-inflammatory cytokines in macrophages that is activated by LPS through toll-like receptor 4 (TLR4), while inhibiting the synthesis of anti-inflammatory cytokines. Therefore, tenascin-C plays a role in regulating the axis of inflammation in LPS-activated TLR signaling [8]. In a mouse sepsis model, LPS treatment enhanced the expression of tenascin-C by macrophages, and was associated with induced proinflammatory cytokines by LPS. [9]. Further, high expression of tenascin-C was observed in lung tissue of a porcine sepsis model, and was associated with pulmonary inflammation [10]. This suggests that tenascin-C may be an inflammation promoting factor for sepsis and its expression may be increased in various tissues.

To date, no studies have reported change of serum tenascin-C levels in sepsis and whether it could reflect disease activity and prognosis. We hypothesize that in sepsis, LPS drives expression of tenascin-C in injured tissues including ECM, which is released into circulation. Therefore, change of serum tenascin-C may reflect the extent of tissue damage, disease severity and prognosis. In this study, we conducted a preliminary clinical study to investigate the association between serum tenascin-C concentration and clinical severity, mortality, and inflammatory response in patients with sepsis. Our study will provide serum tenascin-C levels as prognostic markers for patients with sepsis.

## Methods

### Patients

A total of 167 patients with sepsis were included in this study, who were admitted to the Department of Intensive Care Medicine, Seventh People’s Hospital of Shanghai University of TCM, from July 2016 to December 2017. These patients were diagnosed according to the diagnostic criteria proposed by the ACCP/SCCM Consensus Conference [11]. Sepsis was defined as the presence of microbiologically confirmed infections, acute organ failure, and systemic inflammatory response syndrome (at least two of the following parameters: (1) temperature > 38 °C or < 36 °C; (2) heart rate > 90 beats / min; (3) Respiratory rate > 20 beats/min or PaCO_2_ < 32 mmHg; (4) white blood cell (WBC) count > 12,000 or < 4000 cells/mm^3^, or > 10% immature form). Septic shock was defined as septic patients complicated with refractory hypotension (systolic blood pressure < 90 mmHg), and required fluid replacement or vasoconstrictor to maintain blood pressure. The Sepsis-related Organ Failure Assessment (SOFA) score was used to quantitatively estimate the severity of organ damage in patients. These patients received standard treatment, including antibiotics, mechanical ventilation, and fluid resuscitation. All patients were monitored until they were discharged from the ICU or died, with further follow-up for at least 30 days. The control group included 80 gender- and age-matched critically ill patients without sepsis. This study was approved by the ethics committee of Seventh People’s Hospital of Shanghai University of TCM, and conducted in accordance with the Helsinki Declaration. The patient or family signed an informed consent form.

### Measurement of clinical and laboratory data

Continuous variables were recorded for all septic patients: age, SOFA score, ICU days, serum creatinine, lactic acid, white blood cell (WBC) count, and platelets. The categorical variables of all patients were recorded: gender, site of infection, ventilator application, and septic shock. The end point was 30-day mortality. Blood samples were taken within 24 h of the ICU admission, kept at 4 °C, and centrifuged at 5000 g for 5 min to separate serum, and stored at − 80 °C. Serum C-reactive protein (CRP), interleukin-6 (IL-6) and tumor necrosis factor alpha (TNF-α) levels were determined by enzyme-linked immunosorbent assay (ELISA).

### ELISA for serum soluble tenascin-C

Serum tenascin-C levels in controls and septic patients were measured by ELISA kit (Kamiya Biomedical Company, No. KT-50877, USA), according to the manufacturer’s instructions. The absorbance at 450 nm wavelength was measured by a microplate reader. The serum tenascin-C concentration of each case was determined according to the standard curve established by recombinant tenascin-C at different concentrations.

### Statistical analysis

The data was analyzed using SPSS 19.0 statistical software. Continuous variables were expressed as median (quartile), and categorical variables were expressed in frequency (percentage). Wilcoxon-Mann-Whitney test was performed to compare continuous variables between survivors and nonsurvivors, including age, SOFA scores, ICU time, serum creatinine, lactic acid, WBC, CRP, IL-6 and TNF-α. Chi-square test was performed to compare categorical variables between survivors and nonsurvivors, including gender, site of infection, the presence of mechanical ventilation and septic shock. Spearman’s rank sum test was performed to analyze the correlations between tenascin-C and age, SOFA scores, ICU time, serum creatinine, lactic acid, WBC, CRP, IL-6 and TNF-α. Logistic multivariate regression model was used to analyze the independent contribution of tenascin-C and all other variables to 30-day mortality in patients, expressed as odds ratio and 95% confidence interval (CI). The receiver operating characteristic (ROC) curve was made to determine the difference between serum tenascin-C for survivors and nonsurvivors. Kaplan-Meier curve was plotted to determine the effect of high serum tenascin-C levels (≥56.9 pg/mL) on 30-day mortality in patients with sepsis. *P* < 0.05 was considered as a criterion for significant difference.

## Results

### General characteristics of study population

Of the 167 patients with sepsis admitted to ICU, there were 107 male and 60 female, with a median age of 60 years. The characteristics of septic patients are shown in Table 1. After 30 days of follow-up, 58 patients died and the mortality rate was 34.7%. Patients in the nonsurvivors group had significantly higher age (*P* = 0.024), SOFA scores (*P* < 0.001), serum creatinine (*P* = 0.001), lactic acid (*P* = 0.008), CRP (*P* = 0.010), IL-6 (*P* = 0.010), and TNF-α (P = 0.001) levels compared with survivors. At the same time, the nonsurvivors group had a higher frequency of septic shock and use of mechanical ventilation (Both *P* < 0.05).

**Table 1:** Characteristics of the study subjects

Variable	Sepsis patients *n* = 167	Survivors n = 109	Nonsurvivors n = 58	*P* value
Male sex^a^	107 (64.1%)	70 (64.2%)	37 (63.8%)	0.956
Age (years)^b^	60 (51–67)	57 (48.5–65)	62 (55–69)	0.024
Site of infection^a^				0.241
Pulmonary	111 (66.5%)	68 (62.4%)	43 (74.1%)	
Abdominal	38 (22.8%)	29 (26.6%)	9 (15.5%)	
Other	18 (10.8%)	12 (11.0%)	6 (10.3%)	
SOFA score^b^	12 (10–13)	11 (10–13)	13 (11–14)	< 0.001
Mechanical ventilation^a^	87 (52.1%)	49 (45.0%)	38 (65.5%)	0.011
ICU time (days)^b^	7 (5–10)	7 (5–9)	7 (5–10)	0.573
Septic shock^a^	93 (55.7%)	50 (45.9%)	43 (74.1%)	< 0.001
Serum creatinine (μmol/L)^b^	110 (85–136)	104 (79.5–128.5)	121 (105–146)	0.001
Lactic acid (mmol/L)^b^	2.8 (2.5–3.2)	2.7 (2.5–3.1)	2.9 (2.6–3.3)	0.008
WBC (10^3^ cells/μL)^b^	15 (13–17)	15 (13–17)	15 (13–17)	0.609
CRP (pg/mL)^b^	198 (176–231)	190 (173–225)	207 (187–238)	0.01
IL-6 (pg/mL)^b^	397 (345–465)	388 (327–458)	416 (376–509)	0.01
TNF-α (pg/mL)^b^	41 (36–47)	39 (35–45)	44 (38–50)	0.001

*SOFA* Sepsis-related Organ Failure Assessment, *ICU* intensive care unit, *WBC* white blood cell count, *CRP* C-reactive protein, *IL-6* Interleukin 6, *TNF-α* Tumor necrosis factor-α

^a^Categorical Variable are expressed as frequency (%) and analyzed by Chi-squared test

^b^Continuous variable are expressed as median (25th to 75th percentiles) and analyzed by Mann-Whitney U test

### Elevation of serum tenascin-C was related to the severity of sepsis

**Fig. 1:**
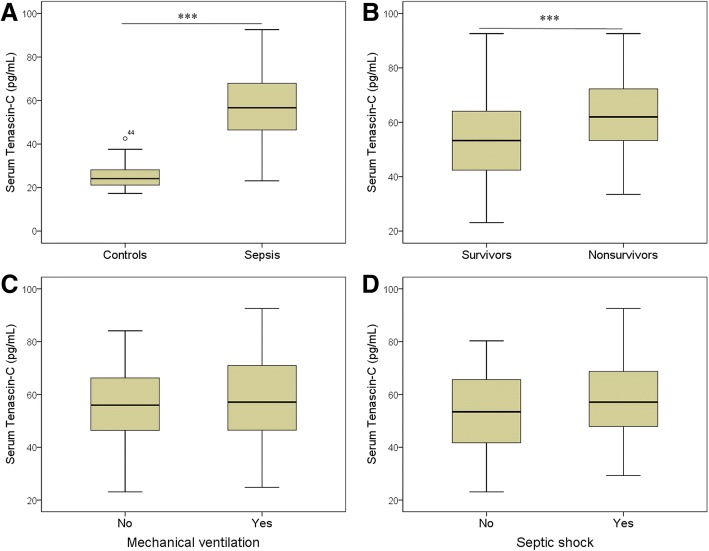
Serum Tenascin-C levels in septic patients and controls. **a** Serum Tenascin-C levels are higher in septic patients (*n* = 167) than in non-sepsis controls (*n* = 80) (*P* < 0.001). **b** Serum Tenascin-C levels are higher in nonsurvivors (*n* = 58) than in survivors (*n* = 109). There are no significant difference in serum Tenascin-C levels between patients with and without mechanical ventilation (**c**), and between patients with and without septic shock (**d**). ***P < 0.001

**Fig. 2:**
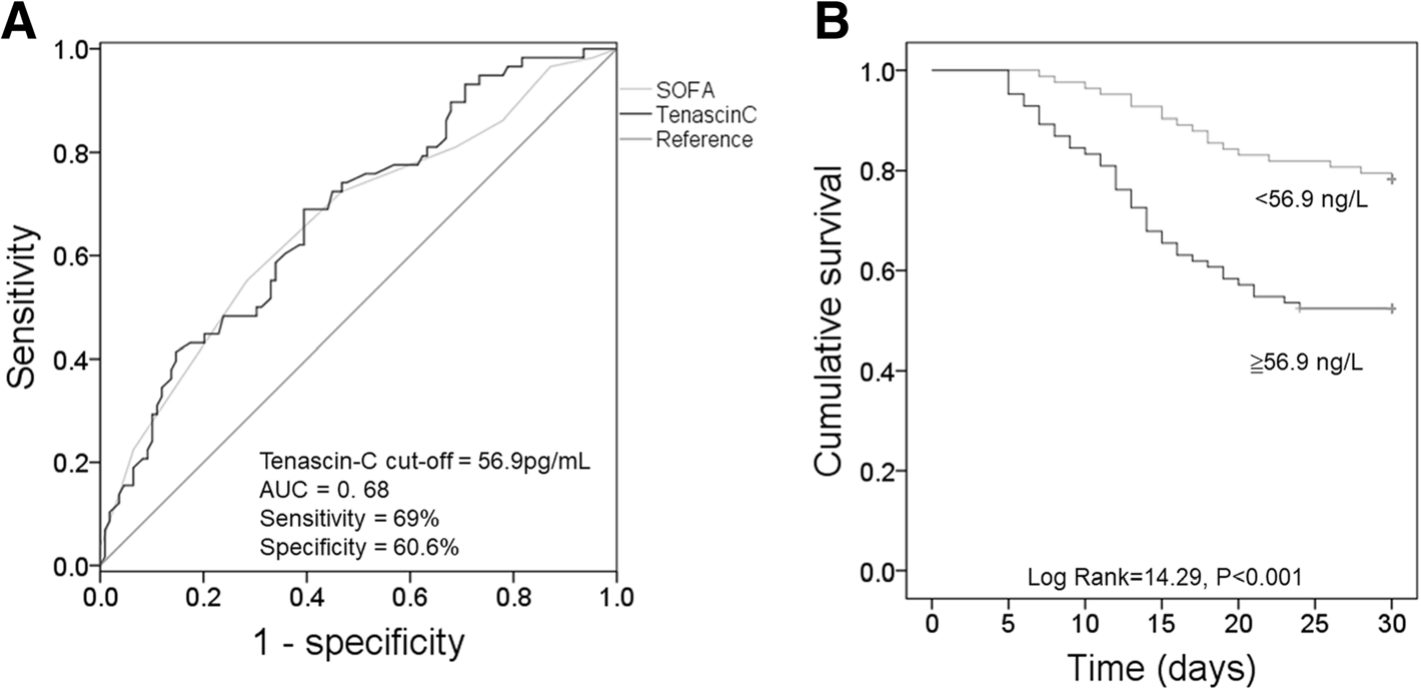
High serum Tenascin-C is closely associated with high mortality in septic patients. **a** The ROC curve is used to determine the cut-off point of serum Tenascin-C that distinguishes between the survivors and the survivors. **b** Kaplan-Meier survival curve shows patients with high serum Tenascin-C (≥56.9 pg/mL) have a significantly elevated 30-day mortality compared to patients with low serum Tenascin-C (< 56.9 pg/mL)

Serum tenascin-C levels (median 56.7 pg/mL) were significantly higher in septic patients than in controls (critically ill patients without sepsis) (median 24.1 pg/mL) (*P* < 0.001) (Fig. 1a). In patients with sepsis, serum levels of tenascin-C were significantly higher in the nonsurvivors (median 64.9 pg/mL) compared with survivors (median 53.3 pg/mL) (*P* < 0.001) (Fig. 2b). There was no significant difference in serum tenascin-C between patients with and without ventilator (Fig. 2c), and between patients with and without septic shock (Fig. 2d).

### Association between serum tenascin-C and clinical variables

We used Spearman rank correlation test to analyze the correlation between serum tenascin-C and continuous variables. In all patients with sepsis, serum tenascin-C was significantly positively correlated with SOFA score (*P* = 0.011), serum creatinine (*P* = 0.006), CRP (*P* = 0.001), IL-6 (*P* < 0.001) and TNF-α (*P* = 0.026) (Table 2).

**Table 2:** Correlation between serum Tenascin-C and other quantitative indicators in patients with sepsis

Variable	Sepsis patients (*n* = 167)	
	r	*P*
Age (years)	0.109	0.162
SOFA score	0.196	0.011
ICU time (days)	−0.038	0.628
Serum creatinine (μmol/L)	0.211	0.006
Lactic acid (mmol/L)	0.071	0.360
WBC (10^3^ cells/μL)	−0.009	0.905
CRP (pg/mL)	0.246	0.001
IL-6 (pg/mL)	0.290	<0.001
TNF-α (pg/mL)	0.172	0.026

r: Correlation coefficient; Test method: Spearman’s rank sum test

### High serum tenascin-C predicts low survival in patients with sepsis

We used Logistic multivariate regression model to investigate the independent contribution of tenascin-C to the prognosis of patients with sepsis. The results showed that serum tenascin-C levels, together with septic shock, lactic acid and TNF-α, were significantly associated with 30-day mortality (Table 3). ROC analysis determined the serum tenascin-C threshold for 30-day mortality. The area under the curve (AUC) was 0.68 (95% CI = 0.597–0.764; *P* < 0.001) (Fig. 2a). The threshold of serum tenascin-C was 56.9 pg/mL (sensitivity was 69%, specificity was 60.6%). We also performed ROC analysis on SOFA score to found the difference between survivors and non survivors. The AUC of SOFA score was 0.666 (95% CI = 0.578–0.754; *P* < 0.001), and the threshold was 12.5 (sensitivity was 55.2%, specificity was 71.6%), this is consistent with those of tenascin-C. We further plotted Kaplan-Meier curve to analyze the effect of high serum tenascin-C on the prognosis of patients with sepsis. Patients with high serum tenascin-C (≥56.9 pg/mL) showed a significantly increased 30-day mortality compared with patients with low serum tenascin-C (< 56.9 pg/mL) (Log Rank = 14.29, P < 0.001) Fig. 2b).

**Table 3:** Logistic multivariate regression predicts 30-day mortality

Variable	Odds Ratio	95% Confidence Interval	*P* value
Septic shock	3.132	1.461–6.713	0.003
Lactic acid (mmol/L)	2.297	1.116–4.725	0.024
TNF-α (pg/mL)	1.049	1.006–1.093	0.026
Tenascin-C (pg/mL)	1.042	1.014–1.071	0.003

## Discussion

In this study, we measured serum tenascin-C levels in 167 patients with sepsis and 80 controls from critically ill patients without sepsis. A significant increase in serum tenascin-C was observed in septic patients compared with controls. In septic patients, serum tenascin-C levels were significantly higher in nonsurvivors compared to survivors. Further analysis showed that serum tenascin-C levels were associated with clinical severity and inflammatory mediators of patients, and acted as an independent prognostic factors. Patients with serum tenascin-C levels ≥56.9 pg/mL had a significantly higher 30-day mortality rate. This article is the first study to investigate the correlation between serum tenascin-C and clinical severity, inflammatory response and prognosis of sepsis.

Tenascin-C is an extracellular matrix protein that is transiently expressed during tissue damage, and its expression reduces to normal level after tissue repair. In addition, persistent high expression of tenascin-C is often present in chronic inflammation [7]. Tenascin-C is not expressed in most adult healthy tissues but is expressed at high levels in infected tissues [12]. Our study found that serum levels of tenascin-C in septic patients were significantly higher than non-sepsis controls, indicating that tenascin-C may be a serum molecular biomarker of sepsis. In LPS-induced septic mice, the expression of tenascin-C by macrophages was significantly enhanced; while treatment with CO inhibited the inflammatory response in septic mice, and also decreased the expression of Tenscin-C induced by LPS [9]. This suggests that tenascin-C may be involved in the pathogenesis of sepsis and become a potential therapeutic target.

We also found that serum levels of tenascin-C were significantly positively correlated with several indicators of sepsis, SOFA scores, and serum creatinine. The SOFA score was used to estimate the severity of sepsis symptoms and multiple organ failure [13], while serum creatinine reflects renal dysfunction in patients with sepsis [14]. Serum tenascin-C is also associated with prognosis in patients with sepsis: serum tenascin-C levels are an independent prognostic factor, and patients with tenascin-C ≥ 56.9 pg/mL have a significantly increased 30-day mortality. Currently, a major challenge for septic patients in ICU is rapid early diagnosis and intervention [15]. Therefore, the search for new biomarkers is the key to achieving this goal and implementing individualized treatment and improving the prognosis of septic patients [16]. Our research indicates that tenascin-C is a protein with multiple biological functions, and may be involved in the pathogenesis of sepsis through numerous unknown mechanisms.

Tenascin-C is a broad inducer of inflammation. High serum levels of tenascin-C are found in many inflammatory diseases such as rheumatoid arthritis [17], collagen disease [18], rheumatoid myocarditis [19] and systemic lupus erythematosus [20]. In acute inflammatory arthritis and knee osteoarthritis, elevated tenascin-C promotes the expression of inflammatory factors and cartilage matrix degradation, both as a marker of joint damage and as a promoter of joint destruction [21]. Our study showed that serum levels of tenascin-C in patients with sepsis were significantly positively correlated with serum inflammatory factors, CRP, IL-6 and TNF-α. After LPS induction, macrophages overexpressed tenascin-C, which enhances the synthesis and secretion of pro-inflammatory cytokines by macrophages, thereby promoting the inflammatory response of Toll-like receptor 4 (TLR4) after LPS induction [8]. Tenascin-C is a broad regulator of the TLR4 axis of inflammation. For instance, in cardiac fibroblasts, tenascin-C upregulates IL-6 expression by activating TLR4 [22]. Tenascin-C and inflammatory factors appear to have a regulatory network. For example, TNF-α can promote the expression of tenascin-C in hepatoma cells and promote metastasis of cancer cells [23]. This self-feedback loop can explain the transient high expression of tenascin-C in acute inflammation and persistently high expression of tenascin-C in chronic inflammation and cancer [24].

The limitations of this study are as follows. Firstly, this is the preliminary result of a study in a hospital that needs to be further confirmed by a multicenter study. Secondly, tenascin-C expression returns to normal after transient high expression in acute inflammation. However, this study only measured the serum level of tenascin-C on admission to hospital in septic patients, and the time series expression of serum tenascin-C and its relationship with prognosis remain further investigation. Thirdly, this is an observational study on the prognostic value of serum tenascin-C, and its clinical significance on diagnostic and predictive values remains unclear. This study only suggests the association between tenascin-C and damage of extracellular matrix (ECM) in sepsis. Fourthly, the source of serum tenascin-C has not been determined. The next study is to measure the tenascin-C expression in peripheral blood mononuclear cells of patients and in macrophage of experimental septic animals.

## Conclusion

This study shows that the concentration of serum tenascin-C in septci patients is elevated. Serum levels of tenascin-C are associated with clinical severity, systemic inflammatory response, and 30-day mortality. Our results suggest that tenascin-C may be involved in the pathogenesis of sepsis and serve as a potential biomarker and therapeutic target.

## Abbreviations

CI: Confidence interval
CRP: C-reactive protein
IL-6: Interleukin-6
LPS: Lipopolysaccharide
MODS: Multiple organ dysfunction syndrome
ROC: Receiver operating characteristic
SIRS: Systemic inflammatory response syndrome
SOFA: Sepsis-related Organ Failure Assessment
TLR4: Toll-like receptor 4 TNF-α
TNF-α: Tumor necrosis factor α
WBC: White blood cell
